# Supplementary material for: ACE-2, TMPRSS2, and Neuropilin-1 Receptor Expression on Human Brain Astrocytes and Pericytes and SARS-CoV-2 Infection Kinetics
Source: Int J Mol Sci. 2023 May 11;24(10):8622. doi: 10.3390/ijms24108622 (PMC10218482; doi:10.3390/ijms24108622)
Supplement: Supplementary file 1 [file ijms-24-08622-s001.zip › ijms-2316847-SI.pdf]

Supplementary Information

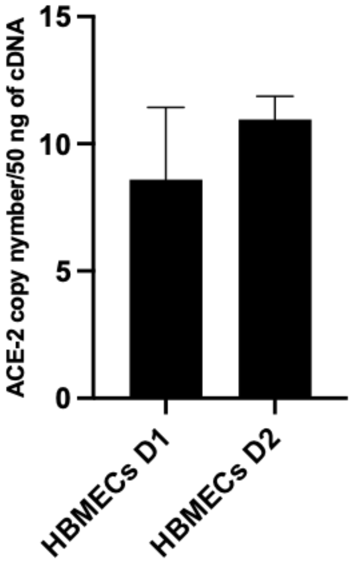

Supplementary Figure S1. ACE-2 gene copy numbers quantitated in 50ng of cDNA of human lymph node derived endothelial cells, (hLNECs), brain derived endothelial cells (hBMVECs) by ddPCR assay.
